# Crystal Structure of *Escherichia coli* Polynucleotide Phosphorylase Core Bound to RNase E, RNA and Manganese: Implications for Catalytic Mechanism and RNA Degradosome Assembly

**DOI:** 10.1016/j.jmb.2009.03.051

**Published:** 2009-05-29

**Authors:** Salima Nurmohamed, Bhamini Vaidialingam, Anastasia J. Callaghan, Ben F. Luisi

**Affiliations:** 1 Department of Biochemistry, University of Cambridge, 80 Tennis Court Road, Cambridge CB2 1GA, UK; 2 Institute of Biomedical and Biomolecular Sciences, University of Portsmouth, Portsmouth, PO1 2DY, UK

**Keywords:** PNPase, polynucleotide phosphorylase (also known as polyribonucleotide nucleotidyltransferase), PNPase core, PNPase ΔKHΔS1 lacking the C-terminal S1 and KH domains, polynucleotide phosphorylase, RNase E, RNA degradosome, RNA degradation, protein-protein interactions

## Abstract

Polynucleotide phosphorylase (PNPase) is a processive exoribonuclease that contributes to messenger RNA turnover and quality control of ribosomal RNA precursors in many bacterial species. In *Escherichia coli*, a proportion of the PNPase is recruited into a multi-enzyme assembly, known as the RNA degradosome, through an interaction with the scaffolding domain of the endoribonuclease RNase E. Here, we report crystal structures of *E. coli* PNPase complexed with the recognition site from RNase E and with manganese in the presence or in the absence of modified RNA. The homotrimeric PNPase engages RNase E on the periphery of its ring-like architecture through a pseudo-continuous anti-parallel β-sheet. A similar interaction pattern occurs in the structurally homologous human exosome between the Rrp45 and Rrp46 subunits. At the centre of the PNPase ring is a tapered channel with an adjustable aperture where RNA bases stack on phenylalanine side chains and trigger structural changes that propagate to the active sites. Manganese can substitute for magnesium as an essential co-factor for PNPase catalysis, and our crystal structure of the enzyme in complex with manganese suggests how the metal is positioned to stabilise the transition state. We discuss the implications of these structural observations for the catalytic mechanism of PNPase, its processive mode of action, and its assembly into the RNA degradosome.

## Introduction

**Fig. 1:**
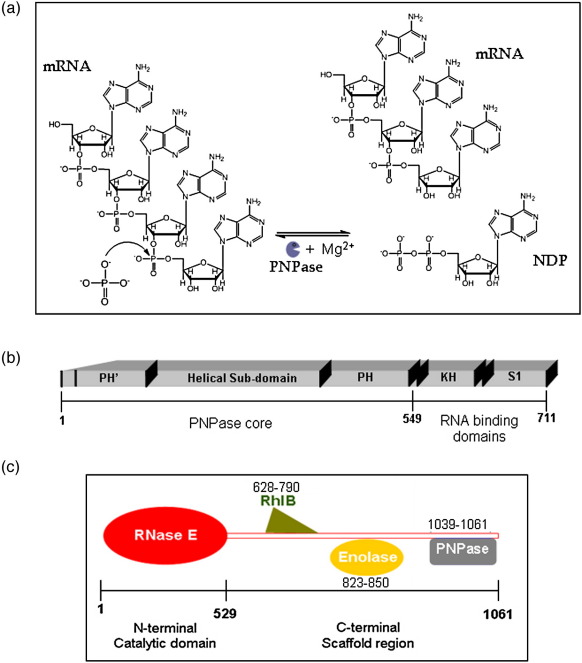
Polynucleotide phosphorylase catalytic activity, domain organisation and interaction site in the RNA degradosome assembly. (a) Phosphorolytic and polymerisation activities of PNPase. Adenine is shown for illustration, but the enzyme can use any RNA base. (b) *E. coli* PNPase domain structure. The key domains of PNPase and the boundaries for the “core” used in this study are shown. (c) Schematic of the canonical degradosome protomer, including the site of interactions of the degradosome components with the C-terminal scaffold region of RNase E. Included in the cartoon are the interaction sites for the DEAD-box helicase RhlB (green), polynucleotide phosphorylase (PNPase, grey), and enolase (yellow). Two RNA-binding sites flank the RhlB interaction region (not shown).

Messenger RNA decay is the counterweight of transcription in the dynamic expression of genetic information. The regulated turnover of transcripts contributes to homeostasis and to timely and efficacious response to environmental change.^1–3^ The biological importance of transcript turnover is suggested by the conservation or essentiality of ribonuclease repertoires in all domains of life. In addition to degrading RNA, many ribonucleases have roles in the maturation and processing of structured-RNA precursors.^4^ One widely occurring ribonuclease with such multi-functional versatility is polynucleotide phosphorylase, a phosphorolytic exoribonuclease (PNPase; polyribonucleotide nucleotidyltransferase; EC 2.7.7.8).^5,6^ PNPase processively cleaves single-stranded RNA substrates in the 3′-to-5′ direction using inorganic phosphate to attack the phosphoester linkage at the 3′ terminus and liberate nucleoside diphosphate (Fig. 1a). Under conditions of excess nucleoside diphosphate and low concentrations of phosphate, PNPase catalyses the reverse reaction to add 3′ extensions to transcripts (Fig. 1a).^7–10^ The length of the extension on transcripts is a likely signature for turnover, since it affects stability and provides a platform for recruitment of PNPase or the hydrolytic exonuclease RNase R.^4^

PNPase is found in diverse species, with homologues identified in eubacteria and eukaryotic organelles such as the chloroplast and mitochondria, although curiously there is no known archaeal PNPase.^8^^,^^11^ In the Gram-negative bacteria *Salmonella* sp., PNPase regulates the expression of small non-coding RNAs that control expression of outer-membrane proteins.^12^ The enzyme also affects complex processes, such as the tissue-invasive virulence of *Salmonella enterica*,^13,14^ and the regulation of a virulence-factor secretion system in *Yersinia*.^15^ In *Escherichia coli*, PNPase is involved in the quality control of ribosomal RNA precursors,^16^ and is required for growth following cold shock.^17,18^

The crystal structure of PNPase from the Gram-positive bacterium *Streptomyces antibioticus* reveals a homotrimeric subunit organisation that encloses a central channel proposed to form the pathway for RNA substrates to enter the active sites.^19^ A duplicated sub-domain within each protomer shares the same fold with the phosphorolytic ribonuclease RNase PH. Although each PNPase protomer potentially contains two active sites, the *S. antibioticus* PNPase structure suggests that only the C-terminal RNase PH domain has the capacity to catalyse phosphorolysis.^19^ The ring-like organisation of the RNase PH-like domains seen in the trimeric PNPase is observed also in the hexameric RNase PH and the hexameric exosome assembly found in eukaryotes and archaea.^8^^,^^20–28^ Crystallographic studies reveal how RNA is accommodated in the central channel of archaeal exosomes.^23–27^ A central channel is seen in the human exosome and is inferred to be present in the yeast exosome, but it is unclear if these engage RNA, as neither the human^25^ nor the yeast exosome^26^^,^^28^ have phosphorolytic activities; instead, the RNase PH-like subunits form a scaffolding support for S1/KH-like RNA-binding subunits and hydrolytic ribonuclease subunits.

Sequence analysis suggests that the duplication of the RNase PH-like domain and the organisation of other sub-domains of *S. antibioticus* PNPase (Fig. 1b) is conserved in PNPases across a wide range of species.^8^^,^^19^ Recent crystallographic analysis of *E. coli* PNPase corroborates the conserved sub-domain organisation.^29^ In both the *S. antibioticus* and *E. coli* crystal structures, the RNase PH-like domains are linked by a helical domain, and there are two RNA-binding domains, S1 and KH, at the C-terminus. The S1 domain is a member of the wider oligosaccharide/oligonucleotide binding fold family (the OB fold) and, like the KH domain, the S1 domain is found in many proteins that interact with single-stranded nucleic acids.^30^ The importance of the S1 and KH domains in PNPase function has been investigated with a truncated version of *E. coli* PNPase lacking both those RNA-binding domains. This truncated PNPase, which we refer to here as the PNPase core, was found to have reduced RNA-binding affinity while retaining phosphorolytic activity.^31,32^ The S1 and KH domains are inferred to interact with a stem–loop structure in the 5′ untranslated region of the PNPase mRNA, since the domains are required for autoregulation of PNPase expression.^18,32–35^ These domains are required also for efficient degradation of short RNA by PNPase.^29^

In *E. coli*, a small proportion of PNPase is associated with the multi-enzyme RNA degradosome (Fig. 1c).^36–38^ The other major components of the degradosome are the endoribonuclease RNase E, the DEAD-box RNA helicase RhlB (EC 3.6.1), and the glycolytic enzyme enolase (EC 4.2.1.11).^36^^,^^39–42^ The C-terminal domain of RNase E is predicted to have little intrinsic structure aside from four recognition segments of increased structural propensity, which are referred to here as micro-domains.^39^ These micro-domains are predicted to recognise the other degradosome components, RNA substrates and the cytoplasmic membrane.^39^^,^^43,44^ Crystallographic studies reveal that an enolase dimer recognises one of the RNase E micro-domains (residues 833–850) by burying the cognate peptide in a deep cleft situated between the enolase subunits.^43^ The recognition site for PNPase has been mapped by deletion analysis^45^ and encompasses a micro-domain in RNase E, corresponding to residues 1021–1061 (Fig. 1c). The corresponding micro-domain has been shown to interact stably with PNPase in solution.^39^

In the current study, we solved several crystal structures of *E. coli* PNPase core bound to its cognate RNase E micro-domain, including a complex with O2′-methyl-modified RNA bound and another with manganese in the active site. We explore salient aspects of the structural data to describe RNA binding, the metal-assisted catalytic mechanism, processivity, and recruitment into the degradosome through micro-domain-mediated recognition.

## Results

### An overview of *E. coli* PNPase core tertiary and quaternary architecture

**Fig. 2:**
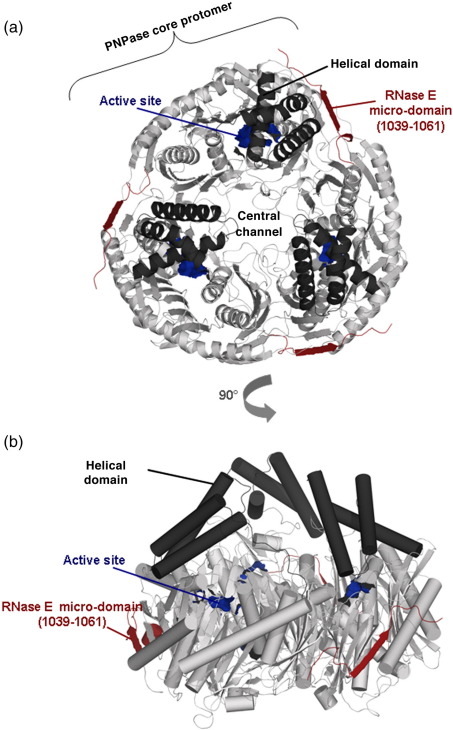

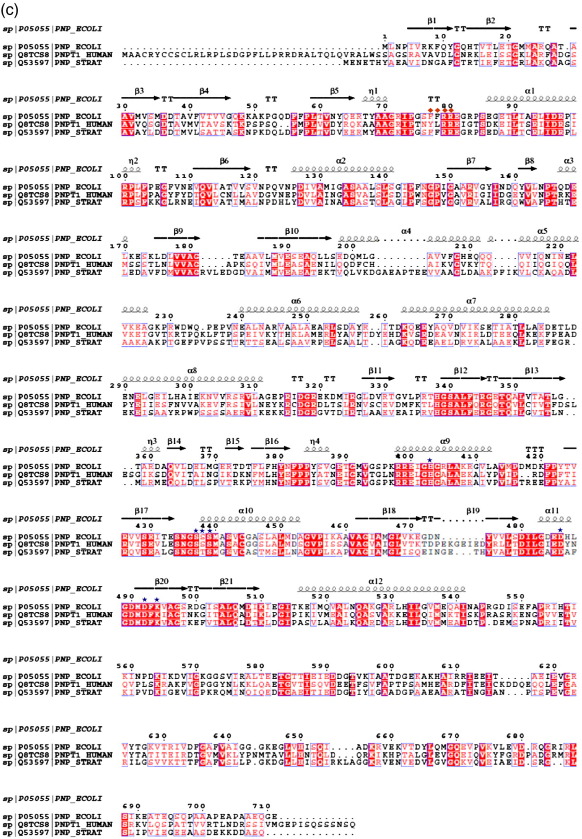
The structure of *E. coli* PNPase core. (a) *E. coli* PNPase core trimer, viewed down the molecular 3-fold axis, showing the central channel, active site (blue) and RNase E micro-domain (red). The helical domain is coloured black (see also Fig. 1b) and the RNase PH subdomains are grey. (b) Rotated view of the PNPase core trimer with the molecular 3-fold axis close to vertical. The secondary structural elements are shown as cylinders and ribbons. In this perspective, the S1 and KH RNA-binding domains, which are not shown, are located on the bottom side of the ring-like trimer. (c) Structure-based sequence alignment of the *E. coli*, *S. antibioticus* and human PNPase core regions. The secondary structural elements of *E. coli* PNPase are shown on the lines above the sequence alignment (except for the S1 and KH domains at the C-terminus). The arrows indicate β-sheet, the coils indicate α-helices, TT indicates β turns and η indicates 3_10_ helices. Red letters indicate homology and blue boxes show similarity. The red highlights indicate identity across the PNPase sequences. The blue stars represent the active site residues and the red diamonds represent the RNA-binding residues in the central channel. Alignments were prepared using SSM (http://www.ebi.ac.uk/msd-srv/ssm), FUGUE, CLUSTALW2 and ProbCons (http://probcons.stanford.edu) and ESPript.^59^ PNPase sequences codes: *E. coli* strain K12, P05055; *S. antibioticus* Q53597; *Homo sapiens* Q8TCS8.

Several crystal forms were obtained for the structures of *E. coli* PNPase core complexed with its recognition micro-domain of RNase E in the presence or in the absence of RNA substrate (Table 1). Subsequent sections will describe interactions with the RNase E recognition micro-domain, metal and RNA, and here we focus on the tertiary and quaternary structure of PNPase itself. Like the *S. antibioticus* homologue,^19^^,^^46^ *E. coli* PNPase core forms a homotrimer with a ring-like architecture that encloses a large central channel (Fig. 2a and b). An aperture is located at one end of this channel where RNA has been predicted to bind.^19^ The two RNase PH-like domains within each protomer are spatially related by a pseudo-dyad axis, so that the PNPase trimer has approximate dihedral symmetry D_3_. *S. antibioticus* and *E. coli* PNPase share 47% sequence identity based on structure alignment (Fig. 2c), and the protomers of their corresponding cores overlay with 1.6 Å root-mean-square deviation from C^α^ positions. The protomer-to-protomer interfaces are similar for the two enzymes, as are the intra-protomer interfaces between the N-terminal and C-terminal RNase PH-like sub-domains (not shown). *S. antibioticus* PNPase has four inter-strand loops that are absent or truncated in the *E. coli* enzyme. Helix H4 of *S. antibioticus* PNPase is absent from the *E. coli* enzyme (Fig. 2c). The *S. antibioticus* and *E. coli* PNPases also differ in details of the packing of the helices in the helical domain that links the two RNase PH-like sub-domains.

The results of recent mutagenesis studies suggest that the helical domain contributes to PNPase enzymatic activity.^47^ The helical domain is well ordered in our RNA-bound crystal structure, where it is seen to form part of a pore that, in addition to the central channel, provides an accessible route to the active site. In the RNA-free form, however, the helical domain is partially disordered, which suggests that it is highly dynamic and may potentially become more structurally defined upon substrate binding. The pore to which the helical domain contributes is unlikely to be a conduit for substrates, since RNA entering through the pore may not have a favourable orientation for the 3′ end to enter the active site. Instead, this helical domain and its associated pore may affect the access of nucleoside diphosphates and phosphate into or out of the active site.

### Recognition of the RNase E micro-domain by the PNPase core

**Fig. 3:**
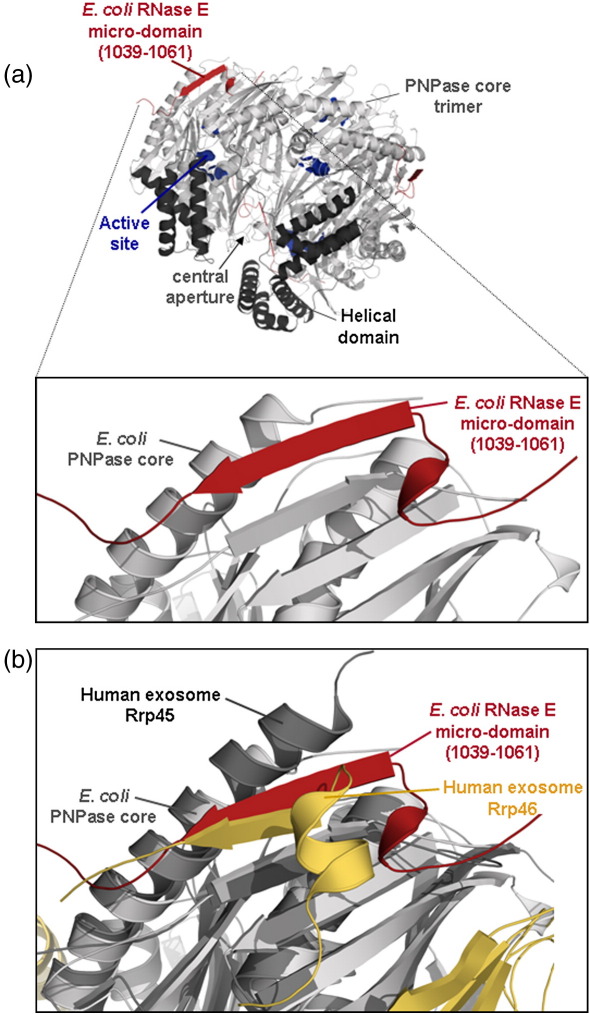
Interactions of RNase E micro-domain and PNPase core. (a) Interaction of the RNase E recognition micro-domain (residues 1021–1061, red) with the solvent-exposed strand of an antiparallel β-sheet of PNPase (grey). The β-sheet is part of the C-terminal RNase PH-like subdomain of PNPase. The view is at the interface of two PNPase protomers, and the perspective is from the S1/KH side of the PNPase ring (i.e., from the bottom of the ring shown in Fig. 2b). (b) Overlay of the *E. coli* PNPase structure (grey) with the Rrp45-Rrp46 in the human exosome (yellow for Rrp46 and black for Rrp 45) showing an interaction for the two exosome subunits that is structurally homologous to that of PNPase core to RNase E.^26^ The perspective is from the same orientation as represented in (a).

PNPase core was co-crystallised with its RNase E recognition micro-domain (residues 1021–1061) under four different conditions, providing several independent views of the micro-domain–enzyme interaction (Table 1). The RNase E micro-domain was well resolved in the tetragonal crystal form, for which interpretable electron density was present for residues 1039–1061 in all three PNPase protomers that occupy the asymmetric unit. The backbone of the RNase E micro-domain forms hydrogen-bonding interactions with the solvent-exposed terminal ridge of an anti-parallel β-sheet within the amino-terminal RNase PH-like sub-domain of the PNPase core (residues 327–331). This interaction generates a pseudo-continuous extended β-sheet (Fig. 2a). The remaining portion of the RNase E micro-domain that is outside the sheet region has a distorted helical conformation (Fig. 3a). The RNase E micro-domain is also well resolved in the rhombohedral crystal form with Mn^2+^ but was poorly resolved in the second rhombohedral form grown in the absence of the metal (Table 1). The location of the micro-domain on the surface of PNPase is corroborated by an orthorhombic crystal form that, while diffracting to only limited resolution (roughly 3.6 Å), has four independent PNPase trimers in the asymmetric unit that reveal unbiased density for 12 copies of the RNase E micro-domain (results not shown).

**Fig. 4:**
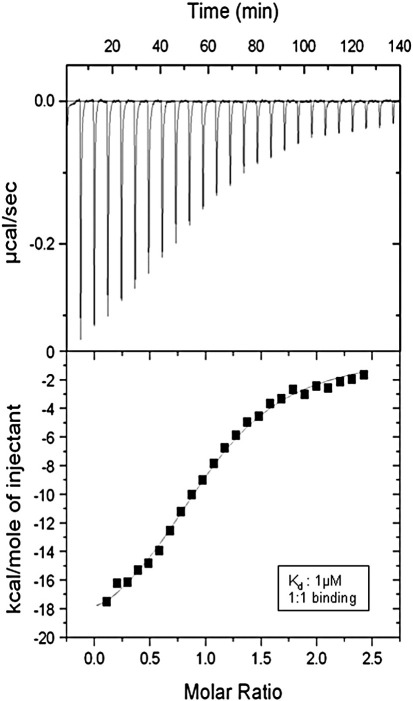
Calorimetric analysis of the interaction of *E. coli* PNPase and RNase E recognition micro-domain (residues 1021–1061). Top panel: The isothermal calorimetry profile showing the heat released upon titrating RNase E micro-domain with PNPase core. Bottom panel: The integrated heats after correction for heat of dilution. The data are best fit with a single binding site model, yielding parameters *N* =  1.02  ±  0.02, *K*_a_ 9.56 ( ±  0.71) × 10^5^ M^-1^, Δ*H* =  –21.48  ±  0.52 kcal mol^–1^, Δ*S* = -44.7 cal mol^–1^ K^–1^.

Because two RNase PH-like domains of the PNPase protomer are related by an internal pseudo-dyad, there are potentially two sites within each protomer that could form an extended sheet-like interaction with the RNase E micro-domain; however, only the amino-terminal RNase PH sub-domain forms an interaction with RNase E. The observed stoichiometry of one PNPase monomer binding to one RNase E micro-domain in the crystal structures is consistent with data from mass spectrometry,^39^ and isothermal titration calorimetry, which show that one RNase E micro-domain binds to each PNPase protomer (Fig. 4). The observed binding affinity by calorimetry is roughly 0.9 μM and is therefore very weak for a macromolecular interaction. The interaction may be stronger in the context of the full-length RNase E, since that molecule is a tetramer and therefore the PNPase binding sites will be spatially co-localised.

An earlier study found that PNPase core and full-length PNPase bind to RNase E with roughly equal affinity.^31^ This suggests that the missing S1 and KH domains do not affect the interaction between RNase E and PNPase, and is consistent with our observation that the bound RNase E micro-domain is not orientated to interact with the S1 and KH domains in PNPase.

Remarkably, the interaction between the RNase E micro-domain and PNPase closely resembles the contact made between the RNase PH-like subunits Rrp45 and Rrp46 in the human exosome (Fig. 3b). A portion of the extended C-terminal tail of the Rrp45 subunit makes a short helical segment and a β-strand that forms an extended sheet with the Rrp46 subunit.^26^ The structural similarity of the *E. coli* RNase E micro-domain-PNPase interaction and the human Rrp45-Rrp46 interaction within the exosome is likely to represent convergent evolution and highlights how the exposed peptide backbone of a β-sheet may be a favoured site for protein–protein interactions.

### A conformational switch accompanies RNA binding at the central aperture

In our screen for well diffracting co-crystals of PNPase core with RNA, several modified RNA oligonucleotides were tested, and well diffracting co-crystals were obtain for a 12-mer RNA in which the sequence originates for a preferred cleavage site for RNase E (Table 1). The RNA is modified with O2′-methyl groups.

**Fig. 5:**
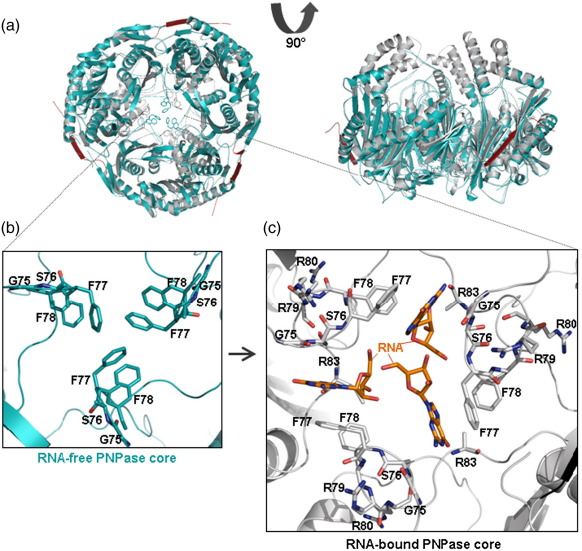

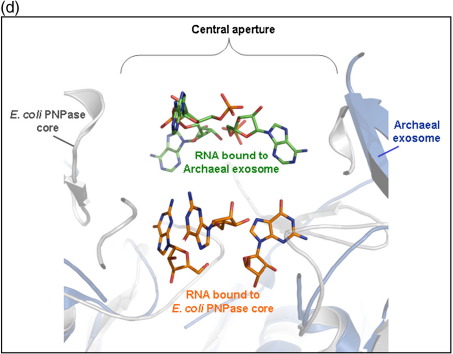
Structural changes associated with RNA binding to the PNPase core. (a) An overlay of the RNA-free (cyan) and RNA-bound (grey) forms of PNPase core viewed down the molecular 3-fold axis (left) and perpendicular to it (right). In the view on the right, the helical domain is on the top of the torus, and the S1 and KH domains (not shown) are on the bottom. (b) Expanded view of the central channel aperture in the RNA-free form. The aperture is occluded by the F77 and F78 side chain of the FFRR loop in this apo-structure, but the loop is less well ordered in the Mn^2+^apo structure and in the apo-structure reported by Shi et al.^29^ (c) The same view as in the left-hand panel but in the RNA-bound form; this shows that the aperture has dilated. It is not clear whether all three RNA-binding sites could be accommodated simultaneously. (d) RNA binds to the central aperture of both *E. coli* PNPase core (orange) and the *S. solfataricus* archaeal exosome (green), albeit at a different depth in the central channel. The view is with the molecular 3-fold oriented vertically.

Previously reported crystal structures of the full-length *S. antibioticus* and *E. coli* PNPases identified two constricted points in the channel.^19^^,^^29^ One of these is closer to the channel entrance and the second is deeper within the channel and nearer the active site. The entrance-proximal aperture is formed by conserved residues, corresponding to *E. coli* residues F77-F78-R79-R80 (Fig. 5a and b). This loop had been predicted to form an RNA-binding site in the PNPases of many species.^19^^,^^46^ Confirming this hypothesis, our structure of PNPase in complex with RNA shows that F77 of each PNPase core monomer makes an aromatic stacking contact with an RNA base (Fig. 5c). The electron density in our structure is not sufficiently resolved to identify the bases, although the shape indicates that they are likely to be purines. F78 of the conserved FFRR loop supports the orientation of the base-contacting F77. The remainder of the RNA away from the stacking contact is disordered and the electron density here is poorly resolved, although it is clear that the pathway followed is along the central pore in the direction of the active site. We do not observe any interpretable electron density at the active site.

Three bases are accommodated with apparent 3-fold rotational symmetry in the channel; however, these bases are likely to originate from three separate RNA oligomers and not from a continuous RNA strand, since the phosphate backbone may not accommodate the required geometry to link the visible nucleotides. Furthermore, it is unlikely that three separate oligonucleotides can be accommodated simultaneously at the aperture due to steric clashes or electrostatic repulsion of the phosphate backbone. We envisage that each potential binding site might be occupied in turn as the oligonucleotide translocates along the pore toward (or away from) the active site.

The stacking of F77 on the RNA base resembles a pyrimidine/purine interaction and covers one face of the RNA base; on the opposite face, the base is contacted by G75 and S76 from the FFRR channel-loop of the neighbouring protomer (Fig. 5c). Thus, binding of RNA requires two channel-loops and may pre-organise one of those to engage another RNA base as the oligonucleotide is translocated along the pore. The corresponding channel-loop in the human PNPase homologue has sequence YLRR, and it is likely to interact with the RNA in the same way as the *E. coli* enzyme (Fig. 2c).

The FFRR channel-loops in the RNA-free *S. antibioticus* PNPase overlap well with the *E. coli* RNA-bound form. However, in one of the RNA-free forms of the *E. coli* PNPase core, the FFRR loop is in a more constricted state (Fig. 5b). In this state, the F77 and F78 of channel-loops of neighbouring protomers stack and partially seal the channel. We note, however, that the FFRR loop may be highly dynamic in the PNPase core, because it is less ordered in the Mn^2^^+^-bound RNA-free structure, and is poorly ordered in the Shi *et al*. structures of the full-length PNPase and the core (PDB codes 3CDJ and 3CDI).^29^ Nonetheless, the aperture becomes dilated and the density becomes well defined in the RNA-bound crystal form, suggesting that the aperture switches conformation upon binding of RNA (Fig. 5b and c). The structural changes propagate to the active site (see the next section), suggesting that RNA binding might help to pre-organise the catalytic centres.

The functional importance of the central aperture residues were verified earlier. Substitution of R79 and R80 in the FFRR loop (R102 and R103 in the numbering scheme used by Shi et al.^29^) to alanine decreased affinity and degradation of RNA substrates,^29^ suggesting that constriction at this point may be involved in RNA capture. Substitution of R80 to aspartate decreases the rates of phosphorolysis and polymerisation roughly 10-fold, showing that the side chain charge is important.^7^ We observe that R79 and R80 do not interact with RNA directly but with other regions of PNPase (Fig. 5c) and suggest that these residues may have a more structural role in contributing to the engagement of RNA by the aperture.

Interestingly, substitution of R83 to alanine (R106 in Ref. 29) had little apparent effect on activity but caused the full-length PNPase to stall on RNA oligomers shorter than eight nucleotides.^29^ This product could span the distance from the aperture to the active site. We observe that R83 is near an RNA base and, although the orientation of the side chain is not clear from the electron density map, it is anticipated to make distributive contact with the phosphate backbone of the RNA (Fig. 5c). The interaction of R83 with RNA may be required for guiding RNA into the active site and perhaps supports a ratchet-like mechanical displacement of substrate into the active site for efficient degradation.

Earlier analyses showed structural similarity of the *S. antibioticus* PNPase and archaeal and human exosomes at the level of protomer fold and quaternary structure.^20^^,^^23,24,27^ Like the *E. coli* PNPase core, the archaeal exosomes engage RNA at the constriction in the central channel.^24,48^ An overlay of the structures of RNA-bound *S. solfataricus* archaeal exosome and *E. coli* PNPase core confirms the expected structural homology. While RNA binds to the central aperture in both bacterial PNPase and archaeal exosome, it is seen at different depths in the channel (Fig. 5d). In comparison to the *E. coli* PNPase core, where the RNA binds towards the internal cavity of the pore as described above, in the archaeal exosome, RNA binds towards the opening of the central aperture *via* a loop region in Rrp41 (residues 62–70), with the key interaction occurring near an incomplete helix (residues 66–69; Fig. 5d). While the RNA bases stack upon a symmetrical ring of phenylalanine residues in the *E. coli* PNPase core, they stack upon histidine residues in the exosome.

### Identification of manganese at the active site

**Fig. 6:**
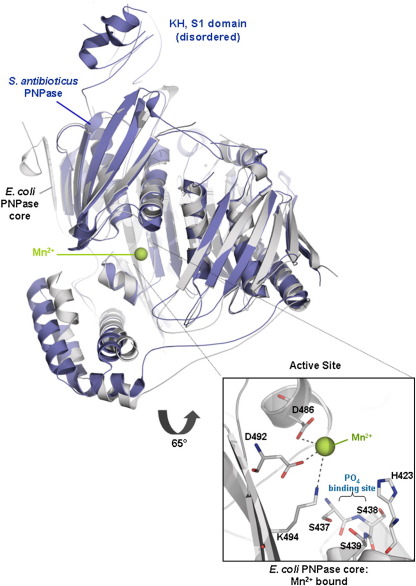
Overlay of the protomers of *E. coli* PNPase core (grey) and *S. antibioticus* PNPase (purple; PDB code 1E3P). The S1 and KH domains, which are disordered, and are shown for the *S. antibioticus* structure.^19,46^ The manganese (green ball) is bound in the *E. coli* active site (inset).

Earlier, the active site of *S. antibioticus* PNPase was identified using the phosphate analogue tungstate, which was found to be coordinated by the side-chain and main-chain atoms of T462 and S463 (corresponding to *E. coli* PNPase residues S438 and S439; see insert of Fig. 6).^19^ The archaeal exosome has a phosphate at the corresponding location.^24^ Magnesium is required for PNPase enzymatic activity, and the metal is expected to be located in the vicinity of the phosphate-binding site. We find that Mn^2+^can substitute for Mg^2+^to support catalysis (S.N. *et al.*, unpublished results), and since Mn^2+^can be more readily identified in difference maps and anomalous Fourier syntheses, we prepared RNA-free co-crystals of the PNPase core in the presence of 20 mM manganese acetate (Table 1). In this crystal form, a single protomer occupies the asymmetric unit, and the map revealed clear density for Mn^2+^at the active site. Mn^2+^is coordinated by the conserved residues D486, D492 and K494 (Figs. 2c and 6, inset), and it is likely that magnesium will bind in a similar manner. The metal-coordinating residues D486 and D492 may act in conjunction with the bound metal to support general acid/base catalysis. Consistent with the proposed metal-binding role of D492, substitution of the residue with glycine abolishes detectable phosphorolysis and polymerisation activities.^7^ These metal-coordinating residues are conserved also in human PNPase (Fig. 2c). The residues corresponding to D486, D492 and K494 are conserved also in RNase PH and the archaeal exosome, and they have been implicated in the catalytic mechanism of *Bacillus subtilis* and *Aquifex aeolicus* RNase PH^21^^,^^22^ and the *Sulfolobus solfataricus* exosome Rrp41 subunit.^27^ Consistent with its role in binding metal, the corresponding site was suggested to hold, at partial occupancy, a cadmium ion originating from the crystallisation buffer in the crystal structure of *B. subtilis* RNase PH.^21^ It seems likely that metal-assisted catalysis is conserved in archaeal exosomes, RNase PH and PNPase.

## Discussion

Our crystallographic studies have explored three salient aspects of PNPase activity and function. The structural data show how PNPase core binds to its cognate RNase E recognition micro-domain; they show how RNA is engaged at a dynamic aperture that gates and orientates substrates to channel along the central pore to the active site; finally, they suggest how the metal-cofactor participates in the catalytic mechanism. We discuss each of these points in turn.

### RNA binding and conformational switching

Our structure of the PNPase core in complex with modified RNA shows that F77 of each PNPase core monomer makes an aromatic stacking contact with an RNA base. F77 is part of the conserved FFRR loop (corresponding to *E. coli* residues 77–80) that forms an aperture at the mouth of the central channel near the S1/KH RNA-binding domains. Mutation of R79 and R80 from this loop affect RNA binding affinity and catalytic activity of PNPase,^29^ and we observe that these residues help to organise the loop to bind RNA. In comparing the RNA-free structures of the PNPase core, we observe that the FFRR loop is dynamic, as we find that it constricts the pore in one apo form, but it is disordered in a second crystal form. In comparison with the constricted state of the apo form, RNA-binding is associated with a dilation of the central aperture to accommodate RNA substrate (Fig. 5b and c). RNA binding is associated with increased conformational order that may be propagated from the aperture to the active site. We observe also that RNA binding at the aperture requires two channel-loops and may pre-organise one of those to engage another RNA base as the oligonucleotide is translocated along the pore. It is likely that the S1 and KH domains may also affect the conformational adjustments, since a comparison of the structures of full-length *E. coli* PNPase and PNPase core in the RNA-free forms shows that the central aperture is expanded in the latter.^29^ As the S1 and KH domains are highly mobile in the crystal structures of both the full-length *E. coli* and *S. antibioticus* PNPase, it is difficult to envisage how these domains might communicate with the core to affect the aperture size. However, it is possible that RNA binding by the KH and S1 domains may itself contribute structural changes at the aperture.

One residue that might be involved in feeding substrate to the active site is R83, which is close to the conserved FFRR aperture loop. While substitution of R83 to alanine (R106 in Ref. 29) has little apparent effect on activity, it causes the full-length PNPase to stall on RNA oligomers shorter than eight nucleotides.^29^ This product could partially span the distance from the aperture to the active site. We observe that R83 is near an RNA base and, although the orientation of the side chain is not clear from the electron density map, it is anticipated to make distributive contact with the phosphate backbone of RNA (Fig. 5c). The interaction of R83 with RNA may be required for guiding RNA into the active site and perhaps supports a ratchet-like mechanical displacement of the substrate into the active site. In summary, we envisage a dynamic aperture at the mouth of the central channel, where binding of RNA pre-organises other potential binding sites on the FFRR loops for the translocating RNA and may pre-organise the active site as well, and in which conserved arginine residues assist the translocation. The potential of the aperture and its neighboring regions to undergo conformational changes to accommodate the RNA is likely to be a key aspect of the process by which PNPase constrains and translocates substrates in its processive mode of action.

### Catalytic role of metal

PNPase and the archaeal exosome Rrp41 subunits catalyse the nucleophilic attack of phosphate on the terminal phosphoester bond and generate nucleoside diphosphate.^51^ Activation is metal-dependent, and the location and activating role of metal has been inferred but not verified experimentally thus far. Our co-crystal structure of *E. coli* PNPase core and Mn^2+^reveals that the metal is coordinated by residues D486, D492 and K494. These residues are also conserved in human PNPase (Fig. 2b). It has been suggested that the residues corresponding to D486 and D492 have roles in the exosome to support general acid-base catalysis,^46^ but we suggest that they are likely to have an additional role in positioning Mg^2+^to activate the phosphate and to support the transition state.

**Fig. 7:**
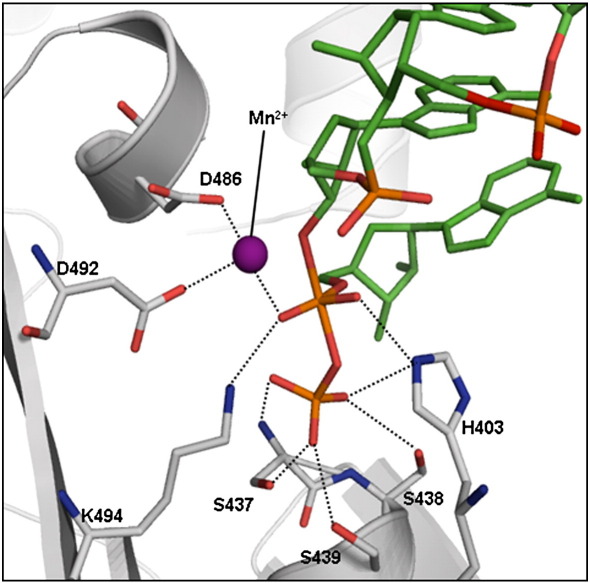
A speculative model showing how metal can interact with the transition state in the *E. coli* PNPase core active site. The text describes the preparation of the model for the transition state using an overlay of ADP and poly(rA) bound structures of the *Pyrococcus abyssi* exosome (PDB codes 2PO0 and 2PO1, respectively).^48^ The β-phosphate of the ADP occupies the binding site for the inorganic phosphate and mimics its orientation in the transition state for either attack of the terminal phosphoester of RNA (for phosphorolysis) or the release of phosphate during polymerisation of NDP. The model was prepared by secondary structure superposition of *E. coli* Mn^2^^+^-bound PNPase structure (grey) with the active subunit of the *P. abysssi* exosome with ADP and poly(rA) bound. The Mn^2+^ (purple ball) coincides almost exactly with the alternative position of the β-phosphate atom in the ADP-bound form.

To examine where the metal might lie with respect to an RNA substrate, we prepared an overlay of the structures of *E. coli* PNPase and *Pyrococcus abyssi* exosome Rrp41 subunit in separate complexes with RNA or ADP (PDB codes 2PO1 and 2PO0, respectively).^46^ Notably, an overlay of the ADP and RNA-bound forms of the *P. abyssi* exosome shows that the scissile phosphate of RNA is spatially coincident with one of two orientations for the α-phosphate of ADP, whereas the β-phosphate is positioned at a binding site for inorganic phosphate. Strikingly, an overlay of ADP and RNA forms resembles the pentavalent phosphate transition state proposed for many phosphotransfer reactions. Guided by this overlay, a model for the transition state of an RNA substrate under attack by PNPase was prepared. Firstly, the phosphate for the transition state was placed at the mean position of the phosphate atoms in the ADP and RNA-bound forms of the *P. abyssi* exosome. Secondly, the oxygen atoms that formed a trigonal planar arrangement were selected from either structure to generate a chimeric structure that resembles the transition state. Lastly, we docked the chimeric RNA structure from the *P. abyssi* exosome structures onto the corresponding position in our PNPase core-manganese structure. The overlay shows that the metal is in a good position to support the proposed transition state (Fig. 7). The metal is well orientated to interact with the carboxylates of D486 and D492, and the pro-chiral, non-esterified oxygens that are in the axial position of the bi-pyrimidal transition state. In the RNA-free structure, the metal is coordinated by water molecules at the positions that correspond to the phosphate oxygens in the transition state. Metal binding is anticipated to be linked favourably with substrate binding, and the metal can have a dual role to support general acid-base catalysis involving protons originating from the water molecules of its hydration shell and to offset charge build-up in the transition state. H403 is predicted to interact with an axial oxygen (Fig. 7), and substitution of this residue with alanine decreases catalytic activity of *E. coli* PNPase 10-fold or greater.^7^ The residues that contact the transition state are conserved in RNase PH and PNPase of all species and in the Rrp41 subunits of the archaeal exosome, suggesting that the mechanism of metal-assisted catalysis is conserved.

### Interaction of PNPase and RNase E, and its implications for the RNA degradosome assembly

Earlier biophysical and computational analyses have indicated that the RNA degradosome is built upon interactions of its canonical components with small peptide micro-domains lying within the C-terminal domain of RNase E.^37^ Our earlier analysis showed that enolase recognises a separate micro-domain within RNase E, and the crystal structure of the complex reveals that the RNase E micro-domain (823–850) is situated in a cleft between the subunits of the enolase dimer,^43^ suggesting the binding of one enolase dimer for every RNase E monomer (S. Nurmohamed *et al.*, unpublished results). Our crystallographic data presented here now reveal how one of these micro-domains from RNase E recognises PNPase core. A segment of 20 amino acids from the RNase E C-terminal domain forms hydrogen-bonding interactions with the exposed ridge of the amino-terminal RNase PH domain of PNPase in an extended β-sheet and also makes van der Waals interactions through a small helical segment. The interaction is at a distance from the active site, and it does not affect enzyme activity (S. Nurmohamed *et al.*, unpublished results).

Remarkably, a similar interaction occurs between the RNase PH-like subunits Rrp45 and Rrp46 in the human exosome, where the C-terminal tail of Rrp46 plays the analogous role of RNase E micro-domain to form a pseudo-continuous β-sheet with the Rrp45 subunit. This similarity is likely to represent convergent evolution and emphasises how the exposed surface of a sheet may be a highly favoured “hot-spot” site for protein–protein interactions. Indeed, molecular recognition through peptide-sheet interaction is seen in several other protein–peptide complexes.^49^ The RNase E homologue of *Streptomyces coelicolor* has been reported to form a stable complex with PNPase^50^ and, although we could not identify a sequence match with the *E. coli* RNase E 1021-1061, we suggest that the interaction of *S. coelicolor* RNase E and PNPase could be mediated by a sheet–strand interaction similar to that used by the *E. coli* proteins.

Using calorimetry, we observe a 1:1 binding stoichiometry of PNPase core to the RNase E micro-domain; moreover, the observed binding affinity is roughly 0.9 μM, which is weak for a macromolecular interaction. The interaction is likely to be stronger in the context of full-length RNase E, which is a tetramer, so that the PNPase binding sites will be concentrated locally. In this regard, it is noteworthy that the observed ratio of 1:1 PNPase:RNase E represents a mismatch of the molecular symmetries of the trimeric PNPase and the tetrameric RNase E. One potential resolution of the mismatch is for the formation of a greater complex of 12 subunits of both proteins, comprising three RNase E tetramers and four PNPase trimers.^42^ Such an arrangement would satisfy all the unique molecular interactions in a self-contained assembly. Nonetheless, it is possible that *in vivo* the degradosomes are not self-contained and are linked together in a more complex incommensurate oligomer. In such a case, the micro-domain of one degradosome is envisaged to interact with an oligomeric partner in a neighbouring degradosome. The PNPase composition of degradosomes isolated from cells can change, depending on physiological conditions,^52^ or in reconstituted degradosomes prepared from recombinant preparations, depending on co-purifying RNA.^53^ We have suggested that additional PNPase beyond the 1:1 stoichiometry might result from shared binding of RNA that links PNPase with the RNA-binding regions in the C-terminal scaffolding domain of RNase E.^5^ Such an arrangement would envisage a dynamic and variable degradosome assembly for which the ancillary binding of additional components, including PNPase, depend on physiological conditions.

## Materials and Methods

### Protein expression and purification

The expression vector encoding *E. coli* PNPase ΔKΔH1 (the PNPase core) was kindly provided by George Mackie (University of British Columbia). The plasmid was transformed into *E. coli* BL21 (DE3), and the core protein expressed by auto-induction.^54^ Cells were cultured at 37 °C in ZYM-5052 medium supplemented with 100 μg/ml ampicillin until an absorbance at 600 nm of 0.6 was reached; the cultures were cooled and grown overnight at 30 °C. After harvesting by centrifugation, the cells were re-suspended in 50 mM Tris–HCl pH 7.5, 150 mM NaCl, EDTA-free Complete Protease tablet (Roche Applied Science) and lysed by repeated passage through an EmulsiFlex-05 cell disruptor (Avestin) until the lysate was free-flowing. Cell debris was removed by centrifugation at 36,000 g, and the supernatant was dialysed into buffer A (50 mM Tris–HCl pH 7.5, 30 mM NaCl, 10% (v/v) glycerol). PNPase core was precipitated from the lysate by incubation at 4 °C in 50% saturated ammonium sulphate. The pellet was collected by centrifugation at 36,000 g, then re-suspended and dialysed against buffer A. The dialysate was fractionated with a Q-Sepharose column (Amersham, Pharmacia) using a salt gradient of 0%–60 % buffer B (50 mM Tris–HCl pH 7.5, 2 M NaCl, 10% glycerol). Fractions enriched in PNPase protein were pooled, dialysed against buffer A and fractionated with a mono-Q 16/10 column (Amersham Pharmacia) using a step gradient to 10 % buffer B, followed by an isocratic gradient at 15 % buffer B. Purified protein fractions were concentrated to 40 mg/mL, dialysed into 20 mM Tris–HCl pH 7.5, 50 mM NaCl and then stored at –80°C.

### RNase E micro-domain and RNA preparation for PNPase co-crystallisations

RNase E micro-domain corresponding to residues 1021***–***1061 was synthesised by Clonestar Biotech Ltd, Czech Republic, desalted using a PD10 column (GE Healthcare), lyophilised, re-suspended in 20 mM Tris***–***HCl pH 7.5, 50 mM NaCl and stored at ***–***80°C. The RNA oligomer 5′-GGGACAGUAUUG-3′ with 5′-monophosphate and O2′-methyl groups was synthesised by Charles Hill (PNAC facility), de-salted, HPLC-purified using an ion***-***exchange column (DNA-pac, Dionex), desalted by preparative G25 chromatography, and lyophilised for storage.

### Co-crystallisations of PNPase core with RNase E recognition site, RNA and manganese

PNPase complexes with RNase E were prepared by mixing 165 μM PNPase core with 246 μM RNase E micro-domain (1021-1061) and kept on ice for 30 min. To prepare the RNA complexes, 330 μM RNA was added to the PNPase/RNase E micro-domain complex and incubated for a further 15 min. Crystals were grown at 20 °C by the hanging-droplet, vapour-diffusion method by mixing 1 μl of complex with 1 μl of reservoir solution. Crystals for the PNPase core/RNase E micro-domain crystals were grown using a reservoir solution containing 0.2 M ammonium nitrate, 20 % (w/v) PEG 3350. Crystals for the PNPase core/RNase E micro-domain-RNA complex were produced from a reservoir solution containing 0.2 M di-ammonium hydrogen citrate, 17% PEG 3350. The measured pH was ∼4.5. The optimal reservoir buffer for the PNPase core/RNase E micro-domain-RNA-tungstate crystals was composed of 0.2 M di-ammonium hydrogen citrate, 17 % PEG 3350, pH ∼4.5, 50 mM disodium tungstate. Crystals for the PNPase core/RNase E micro-domain-Mn^2+^co-crystals were prepared using a reservoir buffer composed of 2.5 M NaCl, 9 % (w/v) PEG 6000, 20 mM sodium citrate and 20 mM manganese acetate tetrahydrate. The crystals were transferred briefly into reservoir solution supplemented with 20–25 % glycerol as cryoprotectant before flash-freezing in liquid nitrogen.

### Data collection, structure determination and refinement

All X-ray diffraction data were collected at 100 K from cryoprotected crystals. X-ray diffraction data for the PNPase/D-micro-domain complex, the PNPase/D-micro-domain–RNA complex and the PNPase–tungstate–RNA complex were collected at the microfocus station ID23-2 at the ESRF facility in Grenoble, France. Data for the manganese crystals were collected with a Raxis IV and Cu rotating anode source. The data were processed and scaled using the HKL package,^55^ and the CCP4 suite was used to convert intensities to amplitudes and for other calculations. Molecular replacement was performed using PHASER^56^ using a monomer from *S. antibioticus* PNPase structure, PDB entry 1E3H.^19^

The refined structure of the *E. coli* PNPase-RNase E micro-domain-RNA complex was used as the search model of the PNPase core/Mn^2+^. The helical domain for this structure (see the domain layout in Fig. 1b) had poorly defined electron density due to disorder, and the side chains and main chain were removed from portions of this domain. Segments of the helical domain were fitted manually and by rigid body refinement, and regions that were not well defined in the electron density at late stages of refinement were removed from the evolving model. The location of the manganese atom at the active site was confirmed by anomalous Fourier synthesis calculated from the Cu-radiation data using unbiased phases and, subsequently, the phases from the refined model. The peak was five standard deviations in height using phases from the refined model. The anomalous map also identified the sulphur atoms of methionine and cysteine residues, and a chloride ion at the helical turn involving the main chain of R66 and the main chain and side chain of T67. Because the refined temperature factors were consistently >80 Å^2^, the occupancy of the Mn^2+^was adjusted to 0.33. The models were built using COOT^57^ and refined using REFMAC5.^58^ A summary of the crystallographic data and refinement are shown in Table 1. Figures of structures were generated with PYMOL†.

### Isothermal titration calorimetry

Isothermal titration calorimetry experiments were carried out at 25 °C using a MicroCal VP-ITC instrument (MicroCal, Northhampton, MA, USA). PNPase core and RNase E micro-domain (1021-1061) were dialysed extensively against 10 mM Tris pH 7.5, 150 mM NaCl, 1 mM MgCl_2_ and filtered with a 0.22 μm pore size filter. Experiments were performed with constant stirring at 280 rpm and each titration consisted of an initial injection of 0.5 μl of PNPase core solution, followed by titrations with increments of 10 μL at intervals of 5 min into a cell containing 1.4 mL of micro-domain solution or buffer control. For all experiments, the PNPase core concentration was 200 μM and the cell concentration of RNase E micro-domain was 10 μM. An identical titration of PNPase core into buffer and buffer into micro-domain were measured to correct the data for the heat of dilution. Data were analysed using MicroCal Origin software version 5.0 and fit with a single binding-site model.

### Protein Data Bank accession numbers

Coordinates and structure factors have been deposited in the Protein Data Bank with accession number 3GCM for the PNPase/RNase E micro-domain/RNA tetragonal crystal form (RCSB ID 051694). The accession number of the PNPase core with RNase E peptide is code 3GLL (RCSB 052013), for the complex with manganese is code 3GME (RCSB 052041), and for the complex with RNA and tungstate is 3H1C (RCSB 052567).

## Figures and Tables

**Table 1:** Crystallographic data^a^ and refinement^b^ summary for PNPase core structures

	PNPase core + RNase E (1021–1061) + Mn^2+^	PNPase core + RNase E (1021–1061) + O2′-methyl RNA 5′-GGGACAGUAUUG-3′	PNPase core + RNase E (1021–1061)	PNPase core + RNase E (1021–1061) + O2′-methyl RNA 5′-GGGACAGUAUUG-3′ + tungstate
Space group	*R*32	*P*4_3_2_1_2	*R*32	*P*2_1_2_1_2_1_
Unit cell dimensions (Å)	*a* = *b* = 158.57, *c* = 156.12, hexagonal setting	*a* = *b* = 176.34, *c* = 189.63	*a* = *b* = 159.15, *c* = 157.51, hexagonal setting	*a* = 167.74, *b* = 262.89, *c* = 264.13
Crystallisation conditions	2.5 M NaCl, 9% (w/v), PEG 6000, 20 mM sodium citrate, 20 mM manganese acetate	0.2 M diammonium hydrogen citrate, 17% (w/v) PEG 3350	0.2 M ammonium nitrate, 20% (w/v) PEG 3350	0.2 M ammonium hydrogen citrate, 17% (w/v) PEG 3350, 50 mM disodium tungstate, pH ∼4.5
Resolution (Å)	20.0–2.40 (2.48–2.40)	20.0–2.50 (2.59–2.50)	20.0–2.70 (2.76–2.70)	79.5–3.57 (3.73–3.57)
Light source, wavelength (Å)	MSC Cu anode, 1.543	ESRF, ID23-2, 0.873	ESRF, ID23-2, 0.873	ESRF, ID23-2, 0.873
No. of unique reflections	27,941	102,846	20,522	129,998
Multiplicity	2.9 (2.8)	6.6 (5.9)	2.9 (2.8)	2.8 (2.4)
Completeness (%)	96.7 (96.7)	99.3 (99.5)	96.7 (96.7)	94.2 (94.2)
*I*/σ	9.2 (1.8)	10.8 (2.2)	9.2 (1.8)	11.4 (2.3)
*R*_merge_ (%)	12.5 (43.2)	14.7 (61.9)	12.5 (43.2)	12.3 (39.2)
Wilson *B*-factor (Å^2^)	48.6	35.1	54.3	31.4
Refinement				
Resolution (Å)	20–2.40	47.3–2.50	20–2.70	25.0–3.57
*R*-factor	0.201	0.208	0.271	0.269
*R*_free_	0.238	0.237	0.309	0.293
No. of reflections used	27,939	97,645	19,414	123,425
Total no. of atoms	3888	13,926	3695	51,235
Total no. of amino acid residues	454	1954	500	6780
Total no. of water molecules, Na^+^, Mn^2+^, Cl^-^, Mg^2+^, citrate	182, 3, 2, 1, 0, 0	955, 0, 0, 0, 3, 8	51, 0, 0, 0, 0, 0	0, 0, 0, 0, 0, 0
Total no. of RNA bases	—	3	—	—

a Crystallographic statistics were calculated by Scalepack, SFCHECK and truncate.

b Refinement statistics were calculated with REFMAC.

## Footnotes

† http://pymol.sourceforge.net
